# Daytime naps improve afternoon power and perceptual measures in elite rugby union athletes—a randomized cross-over trial

**DOI:** 10.1093/sleep/zsad133

**Published:** 2023-05-08

**Authors:** Angus R Teece, Christopher M Beaven, Christos K Argus, Nicholas Gill, Matthew W Driller

**Affiliations:** Te Huataki Waiora School of Health, University of Waikato, Hamilton, New Zealand; Te Huataki Waiora School of Health, University of Waikato, Hamilton, New Zealand; Chiefs Rugby Club, Hamilton, New Zealand; Te Huataki Waiora School of Health, University of Waikato, Hamilton, New Zealand; New Zealand Rugby, Wellington, New Zealand; Sport, Performance, and Nutrition Research Group, School of Allied Health, Human Services and Sport, La Trobe University, Melbourne, Victoria, Australia

**Keywords:** Peak power, team sports, athletic performance, fatigue, exercise exertion

## Abstract

Daytime naps are used by elite athletes in both training and match-day settings. Currently, there are limited interventional studies on the efficacy of napping on physical performance in elite team-sport athletes. Therefore, the objective was to investigate the effect of a daytime nap (<1 hour) on afternoon performance of peak power, reaction time, self-reported wellness, and aerobic performance in professional rugby union athletes. A randomized cross-over design was carried out among 15 professional rugby union athletes. Athletes performed nap (NAP) and no nap (CON) conditions on two occasions, separated by 1 week. Baseline testing of reaction time, self-reported wellness, and a 6-second peak power test on a cycle ergometer were completed in the morning, followed by 2 × 45-minute training sessions, after which athletes completed the NAP or CON condition at 1200 hours. Following the nap period, baseline measures were retested in addition to a 30-minute fixed-intensity interval cycle and a 4-minute maximal effort cycling test. A significant group × time interaction was determined for 6-second peak power output (+157.6 W, *p* < 0.01, *d* = 1.53), perceived fatigue (−0.2 AU, *p* = 0.01, *d* = 0.37), and muscle soreness (−0.1 AU, *p* = 0.04, *d* = 0.75) in favor of the NAP condition. A significantly lower perceived exertion rating (−1.2 AU, *p* < 0.01, *d* = 1.72) was recorded for the fixed-intensity session in favor of NAP.

This study highlights that utilizing daytime naps between training sessions on the same day improved afternoon peak power and lowered perceptions of fatigue, soreness, and exertion during afternoon training in professional rugby union athletes.

## Graphical Abstract

**Graphical Abstract:**
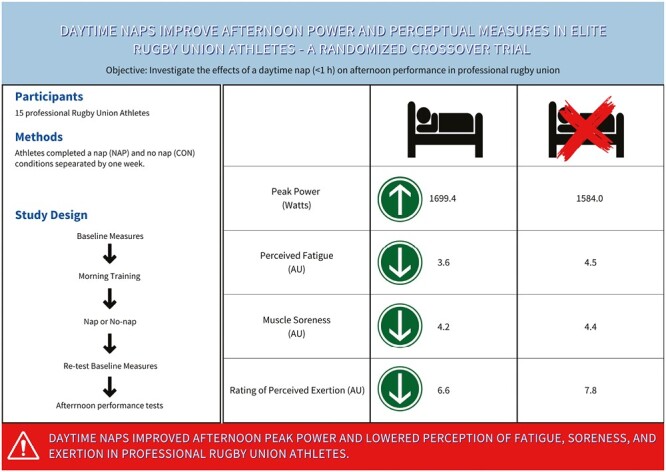

**Statement of Significance**

This study explored the impact of a daytime nap on afternoon performance and perceptual measures of well-being in elite rugby union athletes. Findings highlighted that utilizing a daytime nap of less than 1 hour improved 6-second peak power performance, lowered athletes’ perception of exertion, levels of fatigue, and general muscle soreness in the afternoon compared to when athletes did not take a daytime nap. The findings suggest that using a daytime nap may support recovery between training sessions on the same day and may provide positive effects on afternoon performance amongst elite rugby union athletes.

## Introduction

Insufficient sleep duration has been demonstrated to impact performance, causing decrements in physical performance [1], negatively affecting mood [2], and decreasing cognitive function [3]. Therefore, strategies that can increase total daily sleep may allow for the maintenance of training loads. Extending nighttime sleep has been shown to increase performance, support optimal hormonal responses and adaptation in 25 professional rugby union (rugby) athletes [4]. However, exploring alternative methods to increase total daily sleep is necessary when nighttime sleep cannot be extended due to scheduling constraints. One such method previously used to extend the total daily sleep duration is the use of daytime napping.

Napping has been defined as a period of sleep less than 50% of an individual’s average nocturnal sleep duration [5]. Daaloul et al. [6] investigated the effects of daytime napping to improve performance in elite-level Karate athletes, athletes performed a 30-minute post-lunch nap or post-lunch rest period (no nap) at 13:00 following either a full night’s sleep (>7 hours) or a night of partial sleep deprivation (4 hours). The 30-minute nap enhanced cognitive outcomes following either a full night of sleep or a night of partial sleep deprivation. Furthermore, it was reported that a 30-minute nap had a positive effect on physical and cognitive deteriorations in performance caused by either sleep loss or by fatigue-induced training. Similarly, O’Donnell et al. [7] revealed that a nap of less than 20 minutes enhanced neuromuscular performance. Improvements in countermovement jump peak velocity were observed in favor of the 20-minute nap compared to no nap or a nap longer than 20 minutes in elite netball athletes.

The use of napping in elite athlete populations has been widespread [8], Thornton et al. [9] reported across a 2-week preseason period, 156 naps were taken within a cohort of 31 professional rugby league athletes in both home and away environments. Previous work from our laboratory [10] highlighted that 86% of professional rugby athletes reported napping on a match day throughout the season. Given the widespread use of napping as a strategy in rugby athletes, it seems that this is a population living with sleep debt. Supporting this suggestion, Dunican et al. [11] previously reported that professional rugby athletes obtain an average of 6 hours 30 minutes of sleep per night, which is below the recommended amount of sleep suggested in the general population of 7–9 hours [12]. A lack of sleep in rugby athletes has resulted in symptoms of excessive daytime sleepiness [13] and decreased performance [14]. Extending nighttime sleep may not be possible for professional rugby athletes because of early training [15], playing schedules, and travel requirements [16] and, therefore, may be a reason for the prevalence of daytime naps in this population. There is a paucity of experimental research investigating the effects of daytime naps following morning training on subsequent performance in elite team-sport athletes. Therefore, the objective of this study was to examine the impact of a 1-hour daytime nap on afternoon performance of peak power, reaction time, self-reported wellness, and aerobic performance in a cohort of professional rugby athletes.

## Methods

### Participants

**Figure 1.**
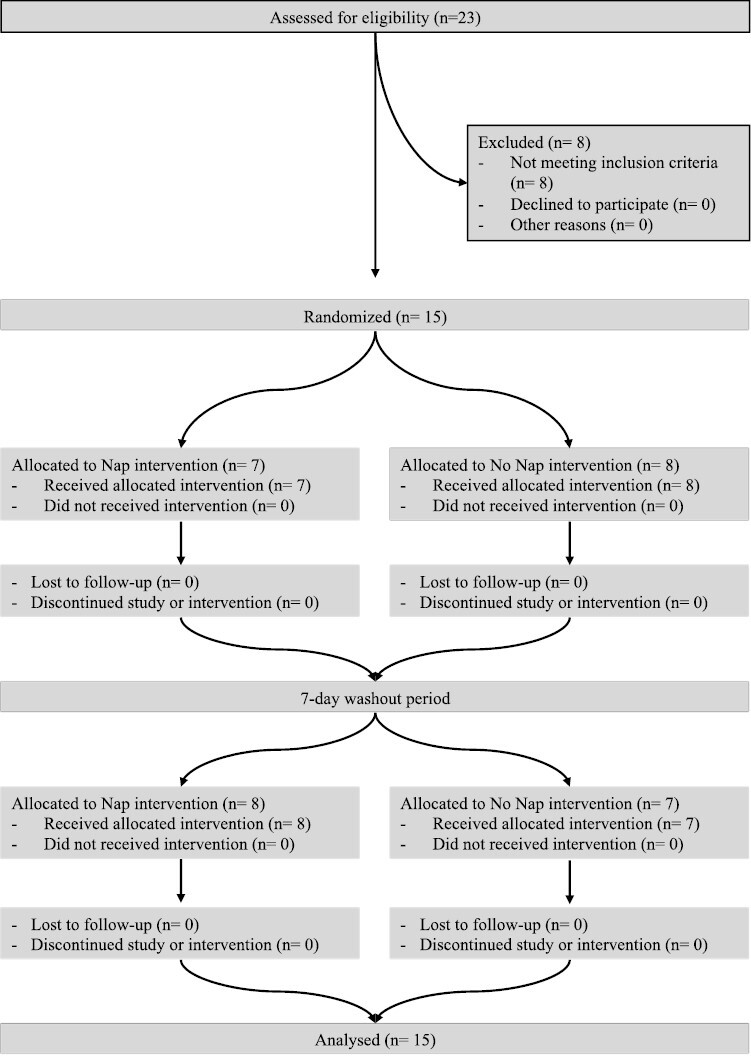
Participant flow diagram.

A total of 23 participants were recruited between April 2019 and July 2019 for the present investigation. To be eligible to participate, athletes were required to be free of illness and injury at the time of data collection, six athletes were excluded from the study due to injury. Additionally, athletes needed to be able to perform both study conditionings over 2 consecutive weeks, which excluded another two from the study (Figure 1.). Therefore, a sample of 15 professional rugby union athletes who competed in the Super Rugby competition (see Table 1 for participant characteristics) participated in the current study, which was conducted in the athletes’ normal training environment. Although 39 athletes were contracted during the time of data collection due to player availability and weekly playing schedules, available participants were limited to 23 athletes, which was therefore the entire population available during the data collection period. Before beginning data collection, the athletes gave their informed consent and ethical approval was granted by the University of Waikato Human Research Ethics Committee in March 2019 (HREC 2019#17).

**Table 1.** Participant Characteristics

Variable	Descriptive statistics	Values
**Age (years)**	Mean ± SD	21 ± 2
**Body mass (kg)**	Mean ± SD	105.7 ± 11.6
**Height (cm)**	Mean ± SD	186.8 ± 5.4

### Research design

**Figure 2.**
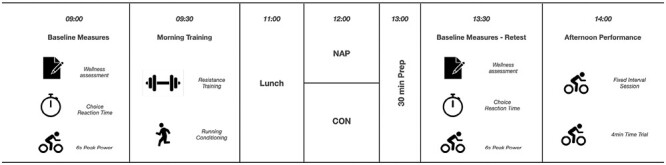
Experimental Protocol.

The study utilized a randomized, counterbalanced, crossover design, as described in Figure 1. Participants were recruited and randomly assigned, according to a computer-generated allocation to a condition by the lead investigator. Participants were assigned to nap condition on testing day one followed by no nap condition on testing day two or non-nap on testing day 1 followed by nap on testing day 2. Each treatment was separated by a 1-week washout period. All training volumes were controlled by the strength and conditioning staff to ensure minimal effects of fatigue between and on the trial days. There were no changes in the protocols after the first participant started the study. A familiarization trial took place prior to the first day of data collection, and all athletes were familiar with all tests and surveys performed. The day before data collection, athletes received a wrist actigraph (ReadiBand, Fatigue Science, Vancouver, BC, Canada.) which they were required to wear the night before data collection to collect sleep measures. On data collection days, athletes were required to undertake baseline measures (self-reported wellness, choice reaction time, and 6-second peak power) followed by two typical training activities of 45-minute duration (resistance training and running conditioning: see below for details). Morning training was followed by an hour period in which athletes were to have lunch, athletes were prohibited from having caffeine or any other stimulants at any stage during data collection days. At 1200 hours, athletes were randomly assigned to nap (NAP) or no nap (CON) conditions. A 30-minute period separated the NAP and CON from afternoon tests, which included a warm-up consisting of 10 minutes of self-selected intensity cycling and dynamic stretching (hamstrings and quadriceps, hip flexors and extensors, calf, and ankle mobility). This 30-minute period post-nap before afternoon testing was included to allow for and minimize sleep inertia based on previous research [8]. Afternoon testing started with retesting of baseline measures and then consisted of a 30-minute fixed-intensity interval session and a 4-minute maximal effort time trial. Between trials, athletes were instructed to maintain their typical sleep habits (Figure 2).

### Nap trial (NAP)

The nap trial involved athletes attending a dedicated sleep room for a period of 1 hour at 1200 hours. The room was dark and temperature-controlled at 18°C, athletes were provided with eye masks, earplugs, and beds in which to sleep. Athletes were not allowed to bring phones or other technology (e.g. smartwatches) into the sleep room. Athletes were required to stay in the sleep room for the entire hour period and were required to relax in the sleep room for the hour if they could not get to sleep. Following the 1-hour nap period, athletes were asked if they napped (yes or no) and were to report their self-estimated nap duration. During the same 1-hour period, the CON condition was instructed not to sleep and not to consume caffeine or any other stimulants. Athletes were otherwise allowed to do as they usually would between morning and afternoon training sessions, including having access to phones and other technology, such as games and music in the team lounge.

### Sleep monitoring

Quantitative sleep measures were collected the night before data collection to ensure sleep the night before each testing day was similar. Sleep measures were collected via wrist actigraphy (ReadiBand, Fatigue Science, Vancouver, BC, Canada). Athletes were required to wear the wrist actigraph on either wrist the night before data collection [17], and athletes were asked to maintain their regular sleep routine. At the beginning of each data collection day, the data were wirelessly downloaded to an iPad and then analyzed using the manufacturer’s online software. The raw activity data were translated into sleep–wake indices, including total sleep time, sleep efficiency (SE%), and sleep latency. The Readiband has been validated against polysomnography (PSG) and has been deemed to be acceptable with approximately 90% agreement in total sleep duration compared to PSG [18]. Furthermore, the inter-device reliability of the Readiband has been shown to have high levels of agreement [19].

### Self-reported wellness

A wellness questionnaire based on the previous work of Hooper and Mackinnon [20] was completed in the morning and afternoon for both nap and CON conditions. The questionnaire comprised of three questions related to fatigue, general muscle soreness, and alertness. All questions were rated on a scale of 1–7 Likert-type scale where 1 represented a good/desirable score (e.g. very fresh) and 7 represented a poor score (e.g. very fatigued). Research from our laboratory has highlighted that the wellness questionnaire displays acceptable day-to-day reliability with an ICC of 0.73 and a TE of 1.63%.

### Reaction time test

Measures of reaction time were collected at 2-time points (morning and afternoon) for each trial. Reaction time was assessed via a choice reaction time test conducted using the Psych Lab 101 application for iPad (Neurobehavioral Systems, San Francisco, USA). The use of mobile devices to assess choice reaction time has been shown to be a valid and reliable way of assessing choice reaction, as described by Burke et al. [21] The choice reaction test required athletes to react to two different stimuli as quickly as possible and touch the corresponding side of the screen. If the athlete saw a red box, they were required to touch the left-hand side of the screen as quickly as possible. If they saw a blue box appear, they were required to touch the right-hand side of the screen. The athletes were required to complete the test, which consisted of 40 trials of each stimulus. The choice reaction test took approximately 3 minutes in total to complete. At the completion of the test average reaction time was recorded by the research team for analysis.

### Peak power assessment

Peak power was assessed in the morning and afternoon of both conditions using the 6-second peak power test on a Wattbike cycle ergometer (Wattbike Ltd, Nottingham, UK). The 6-second peak power test was performed as an assessment of lower-body neuromuscular power. On the initial trial, saddle and handlebar height positions were set up according to athletes’ preferences and were replicated thereafter. Resistance settings for the test were determined according to the recommendations by the Wattbike software based on the athlete’s body mass. The 6-second peak power test was initiated following a 5-second countdown which was followed by a verbal command of “Go.” The test employed a seated stationary start, with the athletes self-selecting the leg which initiated the first downstroke. Athletes were instructed to remain in a seated position and produce maximal effort for the 6-second duration. Peak and relative peak power (W and W∙kg^−1^) were recorded for analysis. The test–retest reliability of the 6-second peak power assessment has previously been reported by Wehbe et al. [22], who reported an ICC of 0.96 and CV of 3.0%.

### Resistance training

Athletes completed a resistance training program which was designed by the team’s strength and conditioning staff. The program was designed to increase maximal strength and power, including three upper-body and three lower-body exercises. The resistance program took approximately 45 minutes to complete, and the same resistance training session was completed for both conditions. At the completion of the resistance training session, athletes were required to report a rating of perceived effort (RPE). Borg’s modified 1–10 scale [23] was used to assess each athlete’s perception of exertion from each training session.

### Conditioning session

Following resistance training, athletes were required to undertake a running conditioning session designed by the team’s strength and conditioning coaches. The session had components of repeated speed, maximal aerobic speed, and high-intensity interval running. The running conditioning session took approximately 45 minutes to complete, and the same session was completed on the same standard-sized rugby field on both occasions. For each session, locomotion activity was measured using an 18 Hz GPS unit to ensure trials were the same each week (Apex Pro Series Pod, STATSports, Belfast, UK). Units were worn on the upper back to decrease variability, and each athlete used the same GPS unit for both conditions. Heart rate (HR) monitors (Polar Electro Oy, Kempele, Finland) were worn by all athletes, with average, and maximal HR recorded by researchers for analysis. Finally, each athlete reported a rating of perceived exertion after the conditioning session. RPE was assessed using Borg’s modified 1–10 scale.

### Fixed-intensity interval cycling test

Athletes undertook a fixed interval cycling test in the afternoon on a Wattbike ergometer on both occasions. The fixed-intensity session was included in the experimental design to ensure that fatigue levels were similar before the 4-minute time trial while also assessing the physiological and perceptual responses to standardized exercise following the NAP and CON trials. Athletes were required to complete a 30-minute interval session which consisted of a 2-minute intensity interval at 2.5 W∙kg^−1^ immediately followed by a 3-minute recovery interval at 1.5 W∙kg^−1^. Athletes completed both intensity and recovery intervals 6 times in total. Resistance settings were self-selected by the athletes to maintain the prescribed power outputs. Athletes wore HR monitors with average and maximal HR collected for analysis. Additionally, average power output (W) and RPE were collected at the completion of the test and were used for analysis.

### Four-minute time trial

A 4-minute time trial was performed on a Wattbike cycle ergometer on both occasions to assess afternoon maximal aerobic performance. Athletes self-selected their resistance settings before the commencement of the time trial. The time trial commenced from a stationary start, with athletes selecting the foot which initiated the first downward stroke. The time trial began following a 5-second countdown followed by a verbal confirmation of “Go.” Athletes were instructed to perform maximally throughout the 4-minute test. Throughout the test, athletes were given verbal encouragement and indicators of time remaining however, athletes were blinded from all performance outcomes. HR was consistently monitored throughout the 4-minute period, with average and maximal HR recorded at the 4-minute mark for analysis. The 4-minute time trial has been shown to have strong inter-day reliability, with Driller et al. [24] reporting an ICC of 0.94, a TEM of 8.8 Watts, and a CV of 2.3%.

### Statistical analyses

All descriptive statistics are reported as means ± standard deviation unless otherwise stated. Statistical analysis was performed on 15 participants using the Statistical Package for Social Sciences (V.27.0, SPSS Inc., Chicago, IL, USA), with statistical significance set at *p* < 0.05 for all analyses. A two-way repeated-measures ANOVA, with 2 (condition: NAP, CON) × 2 (time: PRE, POST) factors, was performed to determine the differences in wellness assessments, reaction time, and peak power. Analysis of the studentized residuals showed normality, as assessed by the Shapiro–Wilk test of normality and no outliers were present, as assessed by no studentized residuals greater than ± 3 standard deviations. The main effects were run to identify where statistically significant differences existed and if there were differences between conditions at the POST time point. Additionally, paired-sample *t*-tests were used to compare HR and RPE in the fixed intensity cycling test, power output in the 4-minute time trial, sleep indices, GPS, and HR data between the NAP and CON for morning training. Effect-size statistics were calculated using Cohen’s *d* and interpreted using thresholds of 0.2, 0.5, and 0.8 for *small*, *moderate*, and *large*, respectively [25]. An effect size of <0.2 was considered *trivial,* and the effect was deemed *unclear* if the 95% confidence interval overlapped the thresholds for both *small* (*d* = 0.2) positive and negative effects.

## Results

Analysis of sleep indices the night before each trial (CON and NAP) revealed no differences (*p* > 0.05) between conditions for any sleep measures, including total sleep time (*p* = 0.95), sleep efficiency (*p* = 0.73), and sleep latency (*p* = 0.92) (Table 2). When morning training data was analyzed, the morning resistance training elicited similar RPE’s (5.3 ± 0.8 vs. 5.3 ± 0.7, *p* = 1.0) for the CON and NAP conditions. Additionally, the running conditioning elicited a similar running distance (3019 ± 56 m vs. 3037 ± 80 m, *p* = 0.58), mean HR (156 ± 7 beats∙min^−1^ vs. 154 ± 6 beats∙min^-1^, *p* = 0.10) and RPE (8.3 ± 0.6 vs. 8.3 ± 0.7, *p* = 1.00) for CON and NAP conditions indicating that the morning exercise was completed at a similar physical intensity for each trial. Analysis of athletes’ responses to the 1-hour nap period revealed all athletes reported being able to successfully nap, with an average self-perceived nap duration of 35 ± 10 minutes.

**Table 2.** Data (mean ± SD) for Comparison of Total Sleep Time, Sleep Efficiency and Sleep Latency as Assessed Via Wrist Actigraphy Between the NAP and CON Conditions From the Night Before Data Collection, Including *P*-values and Effects Size Comparison Between Conditions

Measure	Condition	Mean ± SD	ES (*d)* [95% CI]
**Total sleep time** **(h:mm)**	NAP CON	7:25 ± 0:41 7:23 ± 0:44	0.03 [−0.86, 0.92], *unclear*
**Sleep efficiency** **(%)**	NAP CON	90.0 ± 3.2 89.1 ± 3.8	0.20 [−0.84, 1.24], *unclear*
**Sleep latency** **(min)**	NAP CON	11.1 ± 4.0 10.8 ± 3.9	0.06 [−1.07, 1.19], *unclear*

### Peak power

**Figure 3.**
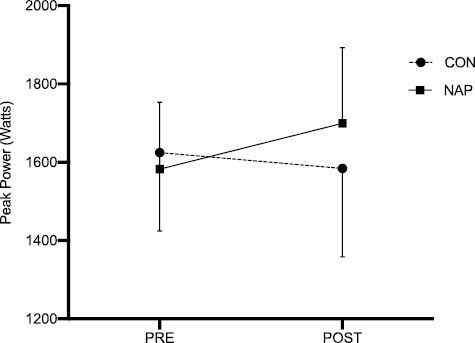
Mean 6 seconds peak power (W) from PRE (morning) to POST (afternoon) timepoints for the NAP and CON trials. Error bars represent standard deviations. * significant group × time interaction.

**Figure 4.**
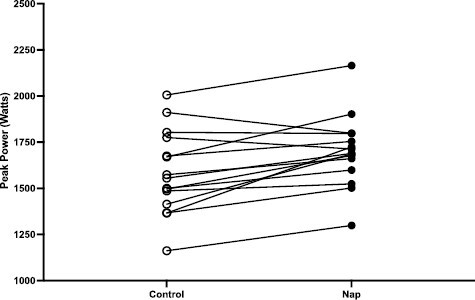
Individual scores of 6 seconds peak power (W) at the POST (afternoon) timepoint for NAP and CON trials.

Results from the 2-way repeated measure ANOVA revealed a statistically significant group × time interaction for absolute peak power, *F*(1,14) = 21.46, *p* < 0.01 (Figure 3) and relative peak power, *F*(1,14) = 24.04, *p* < 0.01. The NAP condition was associated with improvements in absolute and relative peak power (Table 3). The POST time point was associated with significantly higher absolute peak power (*p* < 0.01), a mean difference of 157.6 (W) and relative peak power (*p* < 0.01), a mean difference of 1.1 (W∙kg^−1^) compared to CON (Figure 4).

**Table 3.** Data (Mean ± SD) for Comparison Between NAP and CON Conditions for Morning and Afternoon Peak Power in the 6 Seconds Cycle Test, Reaction Time, Fatigue, General Muscle Soreness, and Alertness Including Effect Size Comparison Between Conditions for Both Time Points (PRE = Morning, POST = Afternoon)

		PRE	POST	Change	ES (*d*) [95% CI]
**Absolute peak power (W)**	NAP CON	1582.5 ± 170.9 1624.8 ± 200.2	1699.4 ± 193.3^*^ 1584.0 ± 225.5	116.8 ± 110.4 ^#^ −40.8 ± 96.5	1.53 [0.78, 2.28], *large*
**Relative peak power (W∙kg** ^**−1**^)	NAP CON	15.0 ± 1.2 15.4 ± 1.3	16.1 ± 1.6 ^*^ 15.0 ± 1.8	1.1 ± 1.0 ^#^ −0.4 ± 0.9	1.54 [0.79, 2.29], *large*
**Reaction time (ms)**	NAP CON	353.6 ± 16.9 351.8 ± 19.1	346.5 ± 19.7 349.1 ± 20.9	−7.1 ± 18.0 −2.7 ± 17.7	0.24 [−0.51, 0.99], *unclear*
** Fatigue (AU)**	NAP CON	3.8 ± 0.7 4.0 ± 1.0	3.6 ± 1.5 ^*^ 4.5 ± 1.0	−0.2 ± 1.4 −0.5 ± 1.2	0.48 [0.11, 0.85], *small*
**General muscle soreness (AU)**	NAP CON	4.3 ± 1.0 3.7 ± 0.9 ^^^	4.2 ± 0.8 4.4 ± 1.4	−0.1 ± 0.9 0.7 ± 1.6	0.75 [0.14, 1.36], *moderate*
** Alertness** **(AU)**	NAP CON	3.7 ± 0.8 3.4 ± 1.1	3.4 ± 1.5 4.0 ± 1.1	−0.3 ± 1.8 0.6 ± 1.6	0.66 [0.02, 1.30], *moderate*

^*^Indicates a significant difference between NAP and CON (*p* < 0.05) at the POST time point.

^#^Indicates a significant difference between pre and post measures (*p* < 0.05),

^^^Indicates a significant difference between NAP and CON (*p* < 0.05) at the PRE time point.

AU, Arbitrary Units

### Reaction time

Results from the 2-way repeated measure ANOVA revealed no significant group × time interaction for reaction time, *F*(1,14) = 0.45, *p* = 0.51 (Table 3.)

### Afternoon performance

Analysis of the fixed intensity cycle session revealed no significant (*p* > 0.05) differences for mean power output (191.6 ± 30.5 W vs. 194.5 ± 24.9 W, *p* = 0.65) or average HR (150 ± 8 vs. 152 ± 10 beats min^−1^, *p* = 0.37) between CON and NAP conditions. However, a significant difference (*p* < 0.01, *d* = 1.75) was observed in RPE scores between conditions, with the NAP condition reporting a lower RPE (6.6 ± 0.7 AU) than the CON condition (7.8 ± 0.6 AU).

Analysis of the 4-minute time trial showed no differences between NAP and CON for relative or mean power output or average HR (Table 4).

**Table 4.** Data (Mean ± SD) for Comparison of Mean Power, Relative Power, Average Heart Rate (HR) and Rating of Perceived Exertion Results from the 4-Minute Time Trial Between NAP and CON Conditions, Including *P*-Value and Effect Size Comparison Between Conditions

Measure	Condition	Mean ± SD	ES (*d*) [95% CI]
**Mean power** **(W)**	NAP CON	316.8 ± 46.8 312.6 ± 41.3	0.09 [−0.15, 0.33], *unclear*
**Relative power** **(W∙kg** ^**−1**^)	NAP CON	3.0 ± 0.4 2.9 ± 0.3	0.11 [−0.15, 0.37], *unclear*
**Average heart rate (beats∙min** ^**−1**^)	NAP CON	163 ± 10 163 ± 9	0.00 [−0.30, 0.30], *unclear*

### Self-reported measures

Results from the 2-way repeated measure ANOVA revealed a significant group × time interaction for fatigue, *F*(1,14) = 5.38, *p* = 0.03 and general muscle soreness, *F*(1,14) = 4.67, *p* = 0.04, but not for alertness, F(1,14) = 3.33, *p* = 0.08. A main effect of time was observed for fatigue and general muscle soreness. The NAP condition displayed significantly lower fatigue scores compared to the CON condition at the POST time point (*p* = 0.01). Conversely, the CON condition displayed significantly lower general muscle soreness scores at the PRE time point compared to the NAP condition (*p* = 0.03). Additionally, a *small* effect was observed for changes in fatigue and *moderate* effects were observed for changes in general muscle soreness and alertness, both in favor of the NAP condition (Table 3.)

## Discussion

The aim of the current study was to examine the effects of a short daytime nap following morning training on afternoon performance in a cohort of professional rugby union athletes. Utilizing a daytime nap of less than 1 hour (average of ~35 minutes) improved afternoon peak 6-second cycling power, lowered afternoon perception of exertion during exercise, and improved afternoon levels of fatigue and general muscle soreness. Therefore, a short daytime nap following morning training may support aspects of physical and perceptual recovery in the afternoon amongst professional rugby athletes and may be a useful strategy to implement in these settings.

While this study is the first to evaluate the effects of a daytime nap on afternoon performance in professional rugby athletes, previous research has evaluated the effects of short daytime naps on physical performance in other cohorts. O’Donnell et al. [7] showed that a 20-minute nap mid-afternoon improved countermovement jump peak velocity amongst elite netball athletes on match days. Additionally, Daaloul et al. [6] reported that a 20-minute nap following sport-specific training that induced fatigue improved squat jump and countermovement jump performance amongst elite-level karate athletes. Our findings support the beneficial effects of a short nap on physical performance, with athletes displaying increased peak and absolute peak power following a short ~35-minute nap. It is likely that the mechanism behind the increase in peak and relative power observed following the nap is multifactorial. Although speculative, one possible mechanism is that elevated cortisol levels that have been observed following a daytime nap [26] may have been associated with better neuromuscular performance [27, 28]. Indeed, the 6-second cycling test relies heavily upon high neuromuscular function for optimal performance [29]. Neuromuscular fatigue has been shown to impair force capabilities, including strength and power [30], altered movement strategies [31], and how team sport athletes produce high-intensity activities [32]. Force capabilities and locomotive movement are important aspects of performance in training and competition among rugby athletes [28]. Therefore, the findings of a nap supporting neuromuscular performance may be important for afternoon training performance in athlete populations.

An additional benefit of daytime naps previously reported is that naps may reduce the perception of effort during exercise. Blanchfield et al. [33] reported that after a 20-minute nap, athletes cited a lower sense of effort via assessment of RPE following a time-to-exhaustion exercise protocol in trained endurance runners. Our results align with previous findings, with athletes reporting lower RPEs for the fixed-intensity cycling session following the NAP condition. The differences observed in RPE may be due to differing levels of physical fatigue between the nap and no-nap conditions. Previous research has proposed that RPE scores during constant exercise are a direct function of workload [34], suggesting that fatigue is a possible stimulus for increases in RPE scores during exercise [34]. Therefore, the finding from the current investigation may indicate that the nap provided improved physical recovery from the morning training session, resulting in lower ratings of exertion in the afternoon.

Measures of fatigue, sleepiness, and alertness have been reported to improve after a daytime nap [35]. We observed that the nap condition displayed significantly lower levels of Self-reported fatigue and a *moderate* reduction in general muscle soreness in the afternoon compared to the no-nap condition, which may support the suggestion that a short daytime nap supports physical recovery. Previous research agrees with the findings from the current investigation; Bourkhris et al. [36] reported that a 40-minute daytime nap resulted in decreased Self-reported fatigue and delayed onset muscle soreness compared to not taking a nap. The finding that a daytime nap reduces fatigue and muscle soreness observed in the current investigation is of importance amongst team sport athletes. Indeed, fatigue has been suggested to impact decision-making amongst athlete populations [37]. Effective and accurate decision-making is important for skill execution and has been suggested as fundamental for success in rugby performance [38]. Furthermore, general muscle soreness has been suggested to negatively impact neuromuscular performance [39] and be linked to measures of decreased performance in rugby [40]. Therefore, it may be suggested that reducing muscle soreness may be important for sporting performance in rugby athletes. Subsequently, these findings are of high relevance and importance amongst professional athlete populations who train more than once per day.

One possible explanation for the current findings of fatigue and general muscle soreness may be linked to levels of cytokines such as interleukin-6 (IL-6) following the nap. Increased levels of IL-6 have been observed following physical exercise [41] and have been associated with muscle damage [42] and heightened sensations of fatigue [43]. Additionally, levels of IL-6 have been shown to be suppressed following a daytime nap [26]. Another plausible factor that needs to be considered is that the daytime nap may relieve sleep pressure and adenosine buildup in the brain, which increases throughout wake periods [44]. Sleep pressure has been shown to increase in the afternoon [45, 46] and has been associated with reductions in alertness and increased fatigue [47]. Furthermore, adenosine buildup inhibits neurotransmitters within the brain, causing an increase in sleepiness and negatively affecting alertness and fatigue [48].

No differences were observed between conditions for reaction time or Self-reported alertness. The lack of change in alertness and reaction time is contrary to previous results by Daaloul et al. [6], who demonstrated improvements in alertness and cognitive performance following a 30-minute daytime nap. The findings in the current investigation may partly be explained by a degree of sleep inertia present following the nap. Sleep inertia has been shown to impair Self-reported alertness and cognitive performance, including reaction time following awakening [49]. Additionally, it has been shown that sleep inertia can impair alertness and cognitive performance for up to 2 hours after waking [50]. Indeed, within the current investigation, we allowed 30 minutes between waking and reaction time testing; while this may have been enough time to overcome sleep inertia, it is possible that sleep inertia was still present 30 minutes post-nap. Furthermore, performance tests are more sensitive to fatigue when longer in duration [51]. The reaction time test took approximately 3 minutes to complete compared to 6 seconds for peak power which may suggest that reaction time was more impacted by potential fatigue present from any remaining sleep inertia.

Given the applied nature of the present study and the difficulty of conducting research on professional athletes, it must be noted that within the current investigation complete case analysis was able to be conducted on 15 athletes meaning that all 15 athletes who started to study completed both conditions which is a strength of the investigation. However, there are several limitations that we must acknowledge within the current investigation. The nap duration and confirmation of being able to nap during the 1 hour were self-reported by athletes, which relied on athletes to assess whether they actually fell asleep, and we were unable to confirm napping via polysomnography (PSG) or any other objective measures. We felt that PSG was not a practically viable option in this setting, as it would take too long to set up in an already short timeframe. We also felt that it would be too unnatural and uncomfortable to use during a relatively short napping period. Alternatively, we considered the use of actigraphy, however, this method has been shown to have some issues in detecting naps in populations where sleep periods contain significant movement [52]^,^ which has been shown to be an issue amongst rugby union athletes [11]. We also acknowledge that sleep inertia following a nap is highly individual [53] and that the 30-minute duration between napping and post-testing measures may not have been enough time for some athletes to wake up and feel alert. However, the time frames used in the current study were designed to replicate the real-world schedule that these athletes follow. It should be mentioned that due to some performance metrics requiring athletes to exert maximally, we cannot discount that this may be influenced by athletes not being blind to which condition they are undertaking, and therefore the nap may have produced some placebo effect as a result. Furthermore, an addition to strengthen the current study design would have been to collect information about athletes' habitual napping and sleep behaviors. This data would enable comparisons between nap duration between habitual and non-habitual nap takers and evaluate if habitual nap takers performed better in the study. Additionally, collecting habitual sleep duration would have highlighted if any athlete was under any form of sleep debt prior to testing days. Given the positive effects of a nap observed in the current investigation, future research should investigate the chronic application of daytime napping in elite sporting populations and evaluate whether daily napping over longer time frames (e.g. 4+ weeks) would be beneficial to physiological adaptations and performance.

## Conclusion

The current study investigated the impact of a daytime nap on afternoon performance and Self-reported measures in professional rugby union athletes. A short nap of around 35 minutes, resulted in an increase in afternoon peak power production, reduced Self-reported fatigue, less general muscle soreness, and lowered perceived exertion during afternoon training compared to not taking a nap between sessions. The findings from the current study suggest that napping may support recovery between training sessions on the same day and may provide positive effects on afternoon performance amongst professional rugby union athletes. Therefore, we are confident that it is unlikely that any harm would be caused to the athletes by implementing napping into their schedule. Given the potential improvements identified in this study, we would encourage practitioners to implement napping into their daily and weekly schedules. Coaches and performance staff should consider allowing time between training sessions on the same day to provide athletes with an opportunity to take a daytime nap to help improve afternoon training performance.

## Data Availability

All data will be made available on reasonable request.
